# *HE4*, A New Potential Tumor Marker for Early Diagnosis and Predicting of Breast Cancer Progression

**DOI:** 10.30699/IJP.2021.135323.2482

**Published:** 2021-06-12

**Authors:** Nazanin Mirmohseni Namini, Alireza Abdollahi, Monireh Movahedi, Amirnader Emami Razavi, Reza Saghiri

**Affiliations:** 1 *Department of Biochemistry, Faculty of Biological Sciences, North Tehran Branch, Islamic Azad University, Tehran, Iran*; 2 *Department of Pathology, Imam Hospital Complex, Tehran University of Medical Sciences, Tehran, Iran*; 3 *Iran National Tumor Bank, Cancer Biology Research Center, Cancer Institute of Iran, Tehran University of Medical Sciences, Tehran, Iran*; 4 * Department of Biochemistry, Pasteur Institute of Iran, Tehran, Iran*

**Keywords:** Breast neoplasms, Gene expression, HE4, WAP four-disulfide core domain protein 2, WFDC2 protein

## Abstract

**Background & Objective:**

This study examined the potential of human epididymis protein 4 (HE4) as a marker in early diagnosis or as a prognostic factor for breast cancer (BC) patients.

**Methods:**

A total of 31 patients diagnosed with BC were enrolled in the study between 2008 and 2018. The mRNA and protein expression levels of HE4 were analyzed by immunohistochemistry (IHC) and real-time polymerase chain reaction (PCR) in the BC tissue and the non-tumoral adjacent tissue. Using ELISA technique, HE4 plasma levels were also measured in 43 BC patients compared to 43 healthy individuals. The correlation between HE4 expression and clinicopathological features was then investigated.

**Results:**

An increase in HE4 expression was observed at mRNA and protein levels in the BC group compared to the control group (*P*<0.01, *P*<0.0001, respectively). In addition, the relative expression of HE4 mRNA in BC patients showed a significant correlation with the differentiation grade of cancer cells (*P*<0.001). Plasma levels of HE4 was also associated with grade (*P*<0.0001), stage, and tumor size in BC patients (for both *P*<0.01). Patients with metastatic BC (*P*<0.01), lymphatic invasion, and lymph node involvement (for both *P*<0.05) showed significantly higher plasma levels of HE4 expression than patients without metastasis.

**Conclusion:**

According to our findings, upregulation of HE4 may be related to invasive BC phenotype. Measuring plasma levels of HE4 could be useful as a screening test in early diagnosis of BC.

## Introduction

Human epididymis protein 4 (HE4), also known as whey-acidic-protein (WAP) four-disulfide core domain protein 2 (WFDC2), was initially cloned as one of four proteins that are strongly expressed in human epididymis (1). Amino acid sequence analysis implied that HE4 belonged to the WAP domain family and contained two WAP domains with four disulfide bonds at the protein core. WAP domain consists of approximately 50 amino acids and 8 cysteine in a conserved arrangement. WAP domain proteins are typically small secretory proteins that exhibit a variety of functions, including growth and differentiation (2). HE4 gene is located on the long arm of the 20q13.12 chromosome as one of 14 homologous genes, a region which, according to the results of cytogenetic studies, is the location of a group of genes for the production of WAP domain proteins (3, 4). P13 (encodes elafin) and secretory leukocyte protease inhibitor (SLPI) are two of the genes present in the region (5) that are co-expressed with HE4 in the upper aerodigestive, reproductive, and urological tracts (4, 6). These proteins have been demonstrated to have anti-inflammatory and anti-microbial activity against gram negative bacteria and viruses (7, 8), as well as a role in cell growth (9, 10) and cell angiogenesis regulation (11). Altered expressions of SLPI and elafin have been identified in several carcinomas, and it appears that these aberrations may play a role in tumor formation, stimulation of metastatic potential, and development of malignant behavior in cancer cells, including breast cancer (BC) (12, 13). In addition, DNA amplification at chromosomal region 20q12-13 has been shown to be common in a number of cancers, especially in BC (14). HE4 complementary DNA also encodes a protein with a sequence similar to that of extracellular protein inhibitors, which appears to play a role in sperm maturation (15). Although the physiological functions of HE4 and its potential role in carcinogenesis have not yet been fully elucidated, these results suggest that based on the similarity of HE4 to SLPI and P13, HE4 may also have carcinogenic and regulatory functions by regulating tumor proliferation and facilitating cancer development. However, the impact of HE4 on the progression of BC has not been determined yet.

In recent years, the number of studies reporting an increase in HE4 expression in various neoplasms, and frequently in the HE4 blood levels, has been increasing. The findings of these studies suggest that the expression of HE4 protein in normal human tissues is inadequate and it is largely limited to the reproductive tracts and respiratory epithelium of the proximal airways (16). Notably, elevated levels of HE4 expression in ovarian carcinoma samples compared with normal ovarian tissue were observed by several studies and the results revealed a significant increase in HE4 gene expression levels when compared ovarian serous carcinoma with other carcinomas. Although lung adenocarcinoma falls in the second place, it has been reported that breast carcinoma also has moderate levels of HE4 expression (17, 18). In fact, HE4 is not restricted to a certain type of tumor and its immunoreactivity has been also observed in other carcinomas. In 2009, the US Food and Drug Administration (FDA) approved HE4 as a useful marker for monitoring ovarian epithelial cancer (19). Based on the reports, HE4, as a serum marker, has even higher sensitivity and specificity than CA125 for early diagnosis of ovarian cancer (20, 21). Altogether, it has been suggested that increased HE4 expression is associated with adverse clinical factors and stimulates a variety of malignant phenotypes, including cell proliferation, cell invasion capacity, and increased tumor growth (22, 23). Finding a proper serum marker for screening and early detection of BC will certainly be beneficial for those who may be at a higher risk to develop cancer. Serum levels of CA125 and CA15-3 are commonly used clinically for this purpose. However, they have insufficient sensitivity and specificity, and low levels of these markers do not exclude the probability of metastatic BC (24). 

Since the number of studies evaluating the association between BC, clinicopathological features, and HE4 expression is very limited, we carried out this research to further evaluate the issue. Therefore, in order to describe HE4 as a plasma marker and to evaluate its potential value in histopathological and serological diagnosis, we examined the levels of HE4 mRNA and protein expression in BC patients compared with control group. We also attempted to examine the association between HE4 expression and clinicopathological features such as grade, stage, metastasis presence, hormone receptor (HR) status, HER2 expression, and P53 mutation status to assess the eligibility of this factor as an early detection tool or a prognostic biomarker in BC.

## Materials and Methods

**Preparation of Tissue Sample**

We conducted a preliminary study to examine patients who had undergone surgery for primary BC between 2008 and 2018 at Imam Khomeini Hospital Complex, (Tehran, Iran). Thirty-one patients were selected for HE4 assessment, which included different stages and grades of BC identified as breast carcinoma by specific pathological tests, nuclear biopsy, and imaging (age range: 32-81 years; mean age (standard deviation) 54 (12)). None of the patients had received any treatments prior to surgery. Following surgery, samples of breast cancer tissue (BCT) and non-tumoral adjacent tissue (NTAT) (normal control) were collected and embedded in paraffin blocks by the Tumor Bank for Hospital’s Cancer Institute Center. Upon histopathological examination of hematoxylin and eosin stained mastectomy or lumpectomy specimens by a pathologist, they were verified as BC cases. More than 90% of tumor tissues were obtained cautiously from non-necrotic areas. Fresh specimens were stored in liquid nitrogen at -180˚C until the examination. Pathological characteristics of patients such as age, tumor size, metastasis, HR status, HER2 expression, and p53 status were gathered from clinical and histopathological records. According to the Helsinki Declaration and the Minnesota Statute for use of medical information in research, only patients who had given written consent to the use of their medical records were included in the study. Patients with renal insufficiency were excluded. The pathological stage was defined based on the eighth edition of the TNM Classification of Malignant Tumors of the Union for International Cancer Control (25). The differentiation grade of cancer cells was determined by a pathologist in accordance with the fourth edition of the World Health Organization (WHO) breast tumors classification (26).

**Preparation of Plasma Samples**

Venous blood samples from 43 patients with confirmed BC were obtained in the pre-operative and pre-treatment period (age range: 31-81 years; mean age (standard deviation) 53.67 (12.29)). A total of 43 blood samples were obtained from age-matched healthy controls (HC) with no history of malignant diseases; the healthy subjects had not received any kind of blood products during the last three years or experienced any inflammatory conditions at the time. The volunteers underwent routine physical and X-ray examination, as well as serum tumor markers, ensuring the absence of malignancy (age range: 33-85 years; mean age (standard deviation) 50.2 (14.65)). Blood samples collected from the fasting participants in ethylenediamine tetraacetic acid (EDTA) containing tubes were later centrifuged at 2000×g for 10 minutes to obtain plasma. All samples were stored at -180˚c until experimental analysis. 

**Relative Expression of HE4 mRNA in Breast Tissue**

*RNA Isolation*

In order to obtain the most reliable and reproducible biological result and to reduce the analytical variability, equal amounts of tissue (100 mg) were used for RNA extraction. Once breast tissues in liquid nitrogen were ground into a fine powder, total RNA of the tissues were isolated using TRIzol™ reagent (Invitrogen, Carlsbad, CA, USA), and the single-step method which relies on Guanidinium thiocyanate-phenol-chloroform RNA extraction and RNA precipitation with isopropanol following centrifugation (27). Prior to the reverse transcription step, extracted RNA were treated with DNase (RNase-Free DNase Set, Qiagen Valencia, CA, USA) to avoid contamination with genomic DNA. Purity measurements were determined using NanoDrop® ND-1000 UV-Vis Spectrophotometer and A_260/280 _ratios between 1.8 and 2 and A_260/230 _ratios between 2-2.2 were considered as pure RNA. Electrophoresis method by 4% agarose gel was performed to inspect the integrity of extracted RNA (28).

*cDNA Preparation and Real-time PCR*

The cDNA synthesis was performed using cDNA synthesis kit (HelixCript™ Thermo Reverse Trans-criptase, Nanoahelix, Yuseong-gu, Daejeon, South Korea) with 1000 ng of total RNA following the manufacturer’s instructions. To inspect the quality of the synthesized cDNA, PCR products were analyzed by agarose gel electrophoresis. An NRTC (No Reverse Transcriptase Control) sample was used to confirm the absence of genomic DNA contamination. Beta-actin housekeeping gene was used to evaluate the efficiency real-time PCR process since it was found not to be affected by HE4 expression (29). Primers were designed for HE4 and Beta-actin, as internal control, using Primer3 software: HE4 forward 5’-CCAGAACTGCACGCAA-GA-3’, HE4 reverse 5’-CGAGCTGGGGAAAGTTA-ATG-3’, Beta-actin forward 5’-GATCAAGATCAT-TGCTCCTCCTG-3’, Beta-actin reverse 5’-CTAGAAG-CATTTGCGGTGGAC-3’. Real-time PCR was carried out using Syber Green method and 2 µL cDNA (100 ng/µL) in a final volume of 25 µL. Each reaction contained 12.5 µL RealQ Plus 2x Master Mix Green with low ROX™ (AMPLIQON, Denmark), 0.5 µL forward primer (10 pmol), and 0.5 µL reverse primer (10 pmol). The amplification was performed by Exicycler™ 96 Real-Time Quantitative Thermal Block (Bioneer, Daedeok-gu, Daejeon, Republic of Korea) under following conditions: incubation at 95˚C for 10 min, 40 cycles of 95˚C for 15 sec; and 60˚C for 60 sec. To evaluate the specificity of real-time PCR reaction, a negative control sample along with a normal human epididymis tissue sample (as a positive control) were co-amplified with the experimental samples. The number of Ct was detected through fluorescent signal. To avoid batch effects, Beta-actin housekeeping gene and HE4 test samples were analyzed simultaneously. The results were standardized by the ∆Ct=Ct _HE4_–Ct _Beta-actin_ formula for both Breast Cancer Tissues (BCT) and Non-Tumoral Adjacent Tissues (NTAT), and the difference of BCT and NTAT was then calculated as ∆∆Ct. Ultimately, HE4 gene expression was presented as the fold change=2^-∆∆Ct^ (Livak method). All reactions were repeated 3 times for each sample, and the mean value was used as the final Ct.

**HE4 Protein Expression**

*Measurement of HE4 Plasma Expression by ELISA*

HE4 plasma levels were measured in 43 BC plasma samples as well as 43 HC plasma samples using human HE4 ELISA kit (XEMA, Moscow, Russia) with 10 pM sensitivity according to the manufacturer’s instructions. The kit dynamic range was 0.15-5 pM. To avoid batch effects, BC and HC plasma samples were analyzed in pairs at the same time. Optical absorption was measured at 450 nm on an absorbance microplate reader (Sunrise™, Tecan, Switzerland).

*Detection of HE4 Tissue Expression and Localization by Immunohistochemical Staining*

To detect HE4 protein expression, 31 BCT along with 31 NTAT samples were used for immunohistochemistry (IHC) analysis. To prepare microscopic slides, tissues were cut into 4 µm sections and incubated overnight at 60˚C. The slides were then dewaxed in xylene and hydrated in a series of decreasing concentrations of ethanol. Antigen retrieval was carried out in sodium citrate buffer (10 mM, pH=6) for 10 min by microwave heating method followed by cooling down at room temperature for 20 min. Thereafter, endogenous peroxidase activity was quenched by 3% hydrogen peroxide in tris-buffered saline (TBS) for 10 min at room temperature. 

Staining with rabbit polyclonal anti-HE4 antibody (ProteinTech®, USA) at 1:100 dilution was performed at room temperature for 90 min. For HE4 detection, slides were incubated for 60 min at room temperature with bovine anti-rabbit HRP-conjugated labeled polymer (DAKO EnVision®+System-HRP, Denmark). To produce color, incubation at room temperature for 10 min was carried out using DAB+Substrate Chromogen System Liquid (DAKO, Denmark). Sections were counterstained with Mayer’s hematoxylin, dehydrated by ascending ethanol concentrations, and then mounted. Negative control was incubated without the primary antibody and normal human epididymis tissue was used as positive control. The presence of brown stained granules on the cell membrane or in the cytoplasm was considered as a positive signal/result. Tissues were scored based on the intensity of staining and the number of positive tumor cells. HE4 staining intensity was scored as follows: 0 (colorless, negative), 1 (faint yellow, weak), 2 (brown, moderate), and 3 (dark brown, strong). The percentage of cellular staining was also scored as follows: 0 (less than 5%), 1 (5-25%), 2 (26-50%), 3 (51-75%), and 4 (More than 76%) in positively stained areas. The two scores were multiplied and the overall score (H-score) reported as follows: 0-2 (negative), 3-4 (+), 5-8 (++), 9-12 (+++). Negative and (+) were defined as low expression, and (++) along with (+++) were classified as high expression.

**Statistical Analysis**

All statistical analysis were performed using SPSS software (Version 20; SPSS Inc., Chicago, IL, USA), and graphs were designed using Graphpad Prism8 (Graphpad Software, La Jolla, California, USA, http: //www.graphpad .com). Independent T-test and Mann-Whitney U Test were used to compare two groups of normally distributed and not normally distributed data, respectively. One-way ANOVA with DunnettT3 post hoc test were used to compare more than two groups of normally distributed, and the Kruskal-Wallis test to compare more than two groups of not normally distributed data. P-value <0.05 was considered to be statistically significant and the symbols were appointed as follows: * for *P*<0.05, ** for *P*<0.01, *** for *P*<0.001, and **** for *P*<0.0001.

## Results

**Fig. 1:**
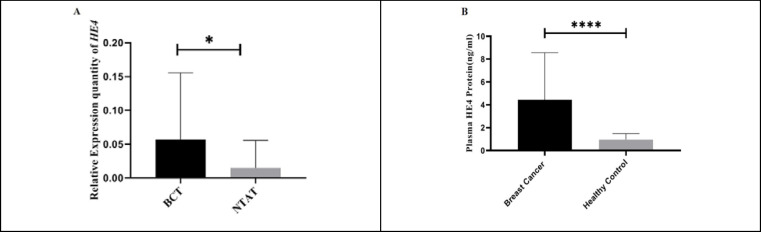
HE4 relative expression in breast cancer tissues (BCT) (n=31) compared with non-tumoral adjacent tissues (NTAT) (n=31). A: The figure shows relative quantification values in 2^-∆Ct ^scale. HE4 mRNA expression in BCT was significantly higher compared with NTAT (*P*<0.05). B: The figure shows higher plasma HE4 levels in BC patients (n=43) compared with healthy volunteers (n = 43) (*P*<0.0001).

HE4 mRNA relative expression was upregulated in BC group compared with HC group. Real-time PCR analysis indicated that HE4 mRNA level in 31 BCT samples were upregulated as compared to NTAT samples. The mean difference (± SEM) was calculated to be -0.041 (± 0.019) between the two groups (*P*=0.004, t (60)= -3.01) (Figure 1A shows the results). 

**Plasma HE4 Expression in BC Patients Increased Compared with Healthy Volunteers**

As shown in Figure 1B, plasma analysis of 43 BC patients by ELISA technique showed a significantly upregulated HE4 level compared with 43 healthy volunteers (*P*=0.0). Table 1 presents descriptive statistical data on the mentioned analysis.

**Table 1:** Levels of HE4 in plasma samples from BC patients and healthy individuals. Mean plasma HE4 values for BC group was significantly higher by 3.48 ng/ml and standard error of 0.63 compared with healthy individuals

		Concentration of HE4 protein in plasma (ng/mL)						
**P-value**	Z	Mean Rank	SD	Median	mean	N		
0.00*	-5.07	57.15 29.85	4.11 0.51	1.98 0.88	4.44 0.96		43 43	**Breast cancer patients** **Healthy individuals**

**HE4 Relative Expression Correlated with the Histological Grade of BC**

**Fig. 2:**
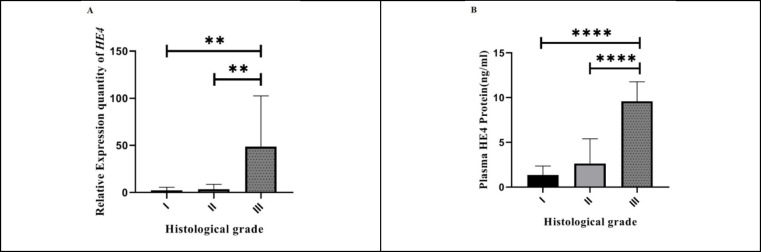
Correlation between HE4 expression and tumor grade. A: The relative expression of HE4 in three grades of breast cancer (Grade I (n = 6), Grade II (n=15), Grade III (n = 9)). As shown, HE4 mRNA expression in grade III was significantly higher compared with grade I (*P*<0.001 for both). B: Correlation between plasma HE4 expression and histological grade. As shown, plasma HE4 expression in grade III (n=13) was significantly higher compared with grade I (n=10) and grade II (n=19) (*P*<0.0001 for both)

We investigated the association of HE4 mRNA expression with tumor grade and stage in BC patients to elucidate whether HE4 expression was correlated with the progression and the grade of differentiation in BC. Table 2 shows the association of HE4 relative expression with the clinicopathological features including age, histological grade, clinical stage, and tumor size. We examined HE4 relative expression in grade I, II, and III tumor samples of BC patients in which significantly higher levels were found for grade III against grade I and II samples (*P*<0.001 for both) (see Figure 2A for more information). While the HE4 expression in the end-stage group (III and IV) was higher compared with the early stage group, the upregulation was not statistically significant. Moreover, HE4 expression in tumor tissue did not show a significant correlation with neither tumor size nor the age of BC patients (*P*>0.05).

**Plasma HE4 Expression in BC Patients Correlated with Grade, Stage, and Tumor Size**

**Fig. 3:**
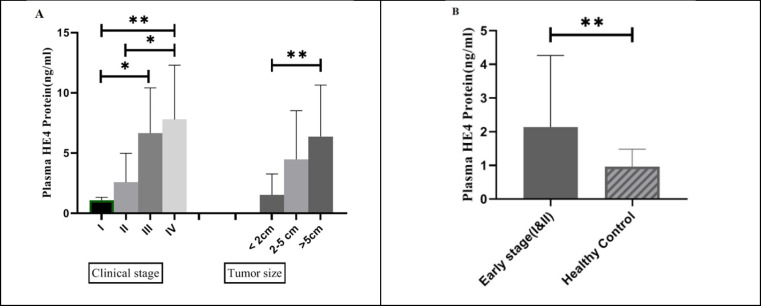
A: Plasma HE4 expression in four stages of breast cancer. A significant increase in plasma HE4 of stage IV (n=12) BC patients was observed compared with stage I (n=7) (*P*<0.01) and stage II (n=17) (*P*<0.05). Plasma HE4 expression was shown to have a significant increase in stage III (n=7) compared with stage I BC patients (*P*<0.05). Also, plasma HE4 expression has a statistically significant correlation with tumor size (*P*<0.01). B: Plasma HE4 expression in early stages of breast cancer (n=24) compared with healthy control group (n=43). The figure shows significant upregulation of plasma HE4 in early stages of breast cancer as compared to healthy individuals (*P*<0.01)

To determine the clinical competence of HE4 expression in BC patients, the correlation between mean values of plasma HE4 expression and clinicopathological features was evaluated by Kruskal-Wallis test (the results are shown in Table 3). A significant difference was observed between plasma HE4 expression and grade (*P*<0.0001) (Figure 2B), stage, and tumor size (*P*<0.01 for both) (Figure 3A) among 43 BC patients. Notably, plasma HE4 expression levels in BC patients did not indicate a statistically significant correlation with age (*P*>0.05). To assess the potential of HE4 as a tumor marker for early screening of BC patients, we evaluated plasma HE4 expression in early stage patients (stage I/ II) against healthy volunteers through which we found a significantly higher plasma expression in early stage BC patients (*P*<0.01) (Figure 3B shows the mentioned results). Table 4 represents the plasma HE4 expression mean values for both groups. 

**Table 2:** Association between HE4 relative expression in BCT samples and clinicopathological features. Results of one-way ANOVA parametric test

Relative expression of HE4								
**P-value**	F	df2	df1	St Error	mean± SD	N (31) (%)		
**0.3**	1.09	28	2	0.34 21.94 4.63	0.86±0.68 30.45±62.06 13.64±20.20	4(12.9) 8(25.8) 19(61.3)	30-40 40-50 >50	**Age(year)**
0.003**	7.451	27	2	1.33 1.39 17.97	2.45±3.27 3.38±5.39 48.69±53.91	6(20) 15(50) 9(30)	I II III	**Grade**
**0.06**	2.68	27	3	0.32 1.57 7.05 15.93	1.06±0.79 4.33±5.23 9.71±12.22 38.46±52.84	6(19.3) 11(35.5) 3(9.7) 11(35.5)	I II III IV	**Stage**
**0.1**	2.183	28	2	0.22 4.16 16.54	1.12±0.66 12.88±13.79 32.23±54.86	9(29) 11(35.5) 11(35.5)	<2 cm 2-5 cm >5 cm	**Tumor size**

**Table 3:** Association between plasma HE4 expression in BC patients and clinicopathological features. Results of Kruskal-Wallis non-parametric test

		Plasma HE4 protein levels (ng/ml)							
**P-value**	Df		Mean Rank	St Error	SD	Mean	N(43)(%)		
**0.1**	2		14.29 25.64 22.56	1.09 1.29 0.82	2.89 4.29 4.11	2.26 6.21 4.28	7(16.3) 11(25.6) 25(58.1)	30-40 40-50 >50	**Age(year)**
0.00****	2		11.80 17.26 35.15	0.32 0.63 0.6	1.02 2.77 2.19	1.34 2.63 9.59	10(23.25) 19(44.18) 13(30.23)	I II III	**Grade**
0.001**	3		10.71 17.35 28.86 31.17	0.1 0.58 1.42 1.29	0.26 2.40 3.77 4.5	1.05 2.57 6.64 7.8	7(16.27) 17(39.53) 7(16.27) 12(27.9)	I II III IV	**Stage**
0.002**	2		12.08 22.08 28.56	0.5 1.12 1.008	1.74 4.03 4.27	1.52 4.58 6.37	12(27.9) 13(30.23) 18(41.9)	<2 cm 2-5 cm >5 cm	**Tumor size**

**Table 4:** Levels of HE4 in plasma samples from BC patients in early stages and healthy individuals. Mean plasma HE4 values for BC patients in early stages was significantly higher by 1.16 ng/mL and standard error of 0.44 compared with healthy individuals

		Concentration of HE4 protein in plasma (ng/mL)					
**P-value**	Z	Mean Rank	SD	Median	Mean	N	
0.003*	-2.97	43.48	2.13	1.13	2.13	24	**Breast cancer patients** **in Early Stages (stages I&II)**
		28.71	0.51	0.88	0.96	43	**Healthy individuals**

**HE4 mRNA Relative Expression is Associated with the Presence of Lymphatic and Vascular Invasion in BC**

To ascertain the association between HE4 mRNA expression with the occurrence of metastasis in BC, we analyzed HE4 relative expression in BC patients (n=31) with and without metastasis. Our results indicated that although HE4 mRNA expression in BC patients including individuals with distant metastases (n=11), perineural invasion (n=12), and lymph nodes metastasis (n=18) were higher compared to the patients without metastasis, evinced upregulation was not statistically significant (*P*>0.05 for all of them). Markedly, HE4 mRNA expression in BC patients with lymphatic invasion (n=20) as well as vascular invasion (n=19) were significantly higher compared to the group without lymphatic/vascular involvement (*P*<0.05 for both) (the results can be seen in Table 5). 

**Plasma HE4 Expression Appears to Be Associated with the Occurrence of Distant Metastasis, Lymphatic Invasion, and Lymph Node Metastasis in BC Patients**

In order to determine the prognostic potential of HE4 as a serological marker, we examined the association of plasma expression with metastasis in 43 BC patients. Our results indicated that plasma levels of HE4 expression in BC patients with distant metastasis (n=12) (*P*<0.01), lymphatic invasion (n=28) and lymph node metastasis (n=25) (for both *P*<0.05) significantly increased compared with non-metastatic BC patients (see Table 6 for the results). 

**HE4 expression is Associated with HR-negative/P53-negative Phenotype in BC**

The correlations between HE4 relative expression and HR status, HER2 expression, and P53 mutation status were investigated in BC patients. As shown in Table 5, HE4 mRNA relative expression was significantly upregulated in HR-negative group (n=16) compared with HR-positive group (n=15) of BC patients (*P*<0.05). Also, BC patients group harboring P53 mutations (n=16) showed higher levels of HE4 relative expression compared with P53-positive group (n=15) (*P*<0.05).

Among 43 BC plasma samples examined, HR-negative (n=20), HER2-positive (n=18), and P53-negative (n=22) phenotypes showed significantly higher levels of HE4 expression compared with HR-positive, HER2-negative, and P53-positive, respectively (*P*<0.01 for all three) (the results are available in Table 6).

**Distant and Lymph Nodes Metastases are Associated with HR Status, HER2 Expression, and P53 Mutation Status in BC Patients**

Spearman correlation analysis of 43 BC patients showed that the incidence of distant metastasis and lymph nodes metastasis significantly increased in HR-negative, HER2-positive, and P53-negative patients (see Table 7 for a better understanding).

**Table 5:** Association between HE4 relative expression in BCT samples and occurrence of metastases, HR status, HER2 expression, and P53 mutation status. Results of parametric independent T-test

Relative expression of HE4						
P-value	T	Df	mean± SD	N (31)(%)		
0.057	2.14	10.15	38.46±52.84 4.16±6.18	11(35.5) 20(64.5)	Yes No	**Distant Metastasis**
0.02*	2.44	19.08	24.44±41.84 1.59±1.47	20(64.5) 11(35.5)	Yes No	**Lymphatic invasion**
0.05	2.1	17.6	25.56±44.08 3.55±4.99	18(58.1) 13(41.9)	Yes No	**Lymph node invasion**
0.02*	2.461	18.06	25.66±42.63 1.57±1.40	19(61.3) 12(38.7)	Yes No	**Vascular invasion**
0.03*	-2.4	15.07	2.21±2.18 29.57±45.55	15(48.4) 16(51.6)	Positive Negative	**ER status**
0.03*	-2.4	15.07	2.21±2.18 29.57±45.55	15(48.4) 16(51.6)	Positive Negative	**PR status**
0.1	1.39	12.55	29.29±50.94 8.15±16.92	12(38.7) 19(61.3)	Positive Negative	**HER-2**
0.04*	-2.144	15.32	3.50±4.65 28.36±46.12	15(48.4) 16(51.6)	Positive Negative	**P53 status**
0.2	-1.106	28	7.83±13.95 22.48±44.21	12(40) 18(60)	Left breast Right breast	**Laterality**

**Table 6:** Association between plasma HE4 expression in BC patients and occurrence of metastases, HR status, HER2 expression, and P53 mutation status. Results of non-parametric Mann-Whitney U test

	Plasma HE4 protein levels (ng/mL)							
**P-value**		Z	Mean Rank	SD	Mean	N(43)(%)		
0.003**		-2.978	31.17 18.45	4.50 3.16	7.80 3.15	12(27.9) 31(72.1)	Yes No	**Distant metastasis**
0.03*		-2.115	24.96 16.47	4.40 2.27	5.64 2.22	28(65.1) 15(34.9)	Yes No	**Lymphatic invasion**
0.01*		-2.53	26.12 16.28	4.43 2.04	6.11 2.12	25(58.1) 18(41.9)	Yes No	**Lymphnode invasion**
**0.07**		-1.81	24.81 17.71	4.47 2.64	5.67 2.58	26(60.5) 17(39.5)	Yes No	**Vascular invasion**
0.001**		-3.23	16.22 28.65	2.33 4.4	2.33 6.88	23(53.5) 20(46.5)	Positive Negative	**ER status**
0.001**		-3.23	16.22 28.65	2.33 4.4	2.33 6.88	23(53.5) 20(46.5)	Positive Negative	**PR status**
0.002**		-3.05	28.89 17.04	4.18 3.30	6.65 2.86	18(41.9) 25(58.1)	Positive Negative	**HER-2**
0.002**		-3.11	15.90 27.82	2.19 4.42	2.23 6.56	21(48.8) 22(51.2)	Positive Negative	**P53 status**

**Table 7:** Correlation analysis of distant metastasis and lymph node invasion, with HR status, HER2 expression, and P53 mutation status in BC patients (n=43). Results of Spearman correlation test

		Distant metastasis		r_s_	P-value	Lymph node Invasion		r_s_	P-value
		M1(n)	M0(n)			Yes(n)	No(n)		
HR status	+ -	0 12	23 8	0.667	0.0****	7 18	16 2	-0.602	0.0****
HER2	+ -	9 3	9 22	0.418	0.005**	16 9	2 16	0.529	0.0****
P53 status	+ -	0 12	21 10	0.608	0.0****	8 17	13 5	-0.397	0.008**

**IHC Staining was Suggestive of HE4 Expression in BCTs**

**Fig. 4:**
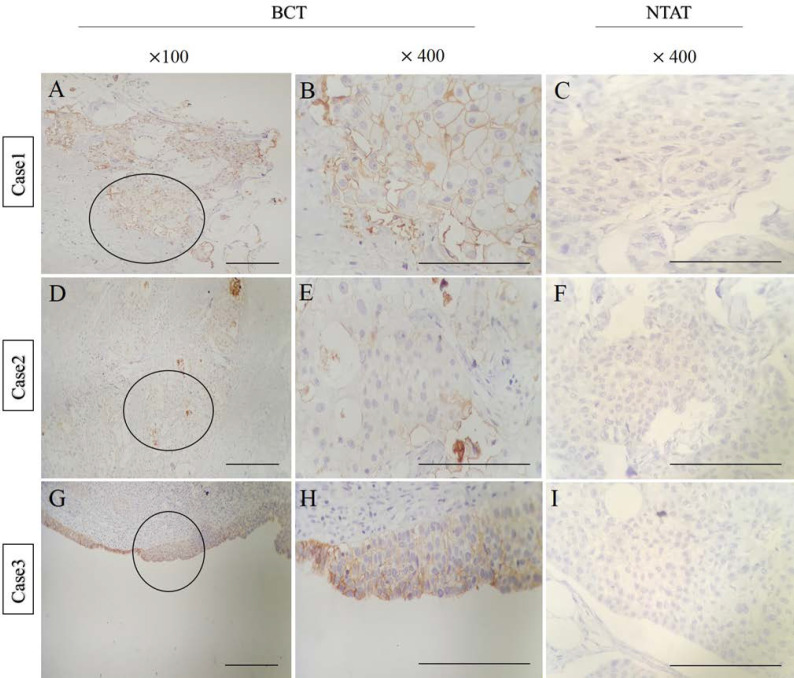
Representative immunohistochemical staining for HE4 in three BCT and NTAT samples. High levels of cytoplasmic expression (++) in BCT samples were detected; A, D, and G: ×100; B, E, and H: ×400. Positive immunoreactivity for HE4 was not observed in NTAT samples; C, F, and I: ×400. (scale bar, 100 µm)

We performed IHC on 31 BCT samples along with 31 NTAT samples to examine whether HE4 was expressed in breast tissue. Three BCT samples (9.6%) were reported as strong positive for the HE4-specific antibody when viewed under a microscope (from 31 total stained: 28 negative cases, 3 positive (++) cases) (see the microscopic results in Figure 4). All 3 positive cases were stage IV BC. HE4 expression was not observed in NTAT samples. Normal epididymis tissue, as a positive control, showed strong staining which confirmed the eligibility of the process. 

## Discussion

There have been significant advances in BC management over the past few decades, which have led to early detection, development of more effective treatments, as well as a significant drop in BC mortality rate (30). Despite improvements in surgical approach and the help of neoadjuvant therapy, many patients are still dying from BC. Thus, it is necessary to find a sensitive and accurate marker for classifying BC patients into different risk groups using clinicopathological features so that patients in lower risk groups do not receive unnecessary treatments, which ultimately reduces the cost and side effects of the treatment.

The product of the *HE4* gene, also known as WFDC2, is a secretory glycoprotein which primarily develops in human ovarian cancer cells with a molecular weight of about 13 KD and further converts to a N-glycosylated protein with a molecular weight of about 25 KD. Several studies suggested that HE4 plays a key role in the diagnosis and monitoring of various cancers, including ovarian and endometrial cancer (31, 32), lung cancer (33), colorectal cancer (34), gastric cancer (35), and BC (36). A number of studies proposed that HE4 has a higher sensitivity compared to CA125 in early detection of endometrial cancer and ovarian cancer in early stages, which indicates the potential of HE4 to predict the recurrence of cancer (37). It has been reported that HE4 can act as a positive regulator in cell adhesion and migration, tumor growth, and cancer metastasis through activating PI3K/AKT and EGFR-MAPK signaling pathways (38, 39). We found that HE4 mRNA expression in tissue of BC patients as well as plasma expression were significantly upregulated compared with HC group. We observed a significant increase in plasma HE4 expression of BC patients in the early stages (stages I and II) compared with healthy individuals; thus, measuring plasma HE4 levels prior to the surgery can potentially discriminate patients and also serve as a serological marker for early detection of primary and/or recurrent BC. Additionally, our results showed that HE4 mRNA and protein expression were increased in both plasma and tumor tissue of high-grade BC; therefore, patients with grade III had the highest HE4 expression level compared with grade I and II. Our findings demonstrated that BC patients with larger-sized primary tumors hold higher plasma HE4 levels than those with smaller-sized tumors. Thus, measuring plasma HE4 expression levels may be useful in pre-operative counseling, decision-making for invasive tumor behaviors, predicting recurrence, evaluation of treatment response, and designing an effective surgery strategy. 

Beyond potential pathological roles, HE4 expression may also be suggestive of the cancer progression from clinical standpoint. Recently, both in-vitro and in-vivo studies have presented that increased HE4 expression is associated with malignant and metastatic characteristics (29, 32). Our results introduced a significant correlation between plasma HE4 levels and adverse prognostic features in BC, which coherently may indicate an association between increased tumor biological invasion and secretion of HE4 in BC. We have shown that HE4 mRNA expression in tissue as well as protein expression in plasma are significantly upregulated in BC patients with distant metastasis, lymphatic invasion, and lymph nodes involvement compared with non-metastatic BC patients. According to the observations, HE4 may increase malignant behaviors in cancer cells such as proliferation, invasion, and metastasis. These findings suggest that HE4 may function as a tumor promoter in BC; however, the mechanisms of function and its biological significance may require further experimental investigation. Consistent with our findings, Kamei *et al.* (40) found that increased HE4 expression is associated with poor prognosis in BC. Also, HE4 can be a predicting marker for lymph node metastasis and may play a key role in cancer recurrence. Researchers also identified genes that were expressed differently in response to HE4, including genes that were involved in mitogen‑activated protein kinase (MAPK) signaling pathways, cell cycle, and DNA-repair mechanisms such as apoptosis regulators (22, 29). These findings suggest that HE4 may perform its biological functions through activating signaling pathways or regulating genes related to growth and apoptosis; however, the significant prognostic value of HE4 is still unknown. 

So far, only few studies have focused on the association of HE4 and hormonal elements. Lokich *et al.* (41) proposed that HE4 interacts with ER-α, which consequently leads to downregulation of ER-α, and thus resistance to anti-estrogens in ovarian cancer cells. Our results consistently showed that HE4 mRNA and protein expression are significantly upregulated in HR-negative BC patients compared with HR-positive individuals. Several studies have suggested that women diagnosed with ER-positive/PR-negative, ER-negative/PR-positive or HR-negative tumors have a higher risk of death compared to women with HR-positive tumors, and that is largely independent of demographic and clinicopathological features of tumors (42). Based on these results, it can be inferred that high levels of HE4 expression may be associated with a higher risk of mortality as well as poor response to treatment. We also found that patients with HER2-positive BC had significantly higher levels of plasma HE4 expression compared to HER2-negative patients. Akoz *et al.* (43) reported that HE4 expression is strongly associated with tumor histological grade and HER2 proliferation, which is consistent with our results. These results may strengthen the hypothesis that HE4 expression increases in accordance with HER2 proliferation in patients. Since, HER2 proliferation is known to be a factor related to poor prognosis in BC, it can be concluded that high levels of HE4 expression may also play a role in inaccurate prognosis. Our results also demonstrated that HE4 expression in tissue and plasma of P53-negative BC patients significantly increases compared with P53-positive patients. Our observations also confirmed that the possibility of distant metastasis and lymph node invasion in BC patients significantly increases in HR-negative, HER2-positive, and P53-negative phenotype. Thus, patients with increased HE4 expression, whose tumors were also HR-negative, HER2-positive, and P53-negative experienced a more invasive disease, which can lead to BC with more malignant behaviors and a poor response to treatment.

According to the IHC results of the present study, in three out of 31 BCT samples (9.6%), a strong positive cytoplasmic staining was observed with HE4 specific antibody. This implied that HE4 protein was also expressed in the cytoplasm of breast tumor cells, which is consistent with the results of other studies (18, 40). However, in a study conducted by Drapkin *et al.* (16), HE4 expression was not detected in breast carcinoma tissue. Also, 26 samples (83.8%) showed higher expression of HE4 mRNA expression in BCT compared with NTAT; but only 9.6% of BCT samples showed HE4 protein expression. The lack of HE4 protein expression in BCT samples on which IHC were performed could be associated with intracellular factors including challenges of protein measurement and technical issues. 

Contrary to previous studies, which demonstrated that normal breast tissue cells (especially ductal cells) had a weakly positive HE4 expression (18, 40), in the present study no expression of HE4 protein was detected in NTAT samples. The staining intensity was not related to grade or stage of the tumor, and all three HE4-positive cases were at stage IV. In addition, all three tissue samples had equivalently strong HE4 expression. However, it should be noted that IHC staining results were only positive in three out of 14 end-stage patients (stage III and stage IV); hence, HE4 expression in BCT should be further studied in order to elucidate the association of HE4 expression and the progression of BC. Since IHC staining varies in breast tumor tissue, absence of staining in a limited biopsy sample should be interpreted with caution.

Our study supported the more extensive use of HE4 in the clinical setting. More quantitative tests should be generated based on plasma to assess HE4 sensitivity in pre-operative and post-operative settings to enable us evaluate the potential of HE4 as a marker in monitoring early stages of breast carcinoma. Further serological tests measuring plasma HE4 in patients with benign and malignant breast diseases are needed. Also, understanding the expression patterns of HE4 could be useful in the evaluation of breast carcinoma as well as histopathological diagnosis. Since HE4 is a secretory glycoprotein, it can also be filtered by the kidneys into the urine, and consequently HE4 may be introduced as a feasible target for generating a cancer screening urine test.

We confirm that the findings of the present preliminary study do not provide complete evidence on the predictive value and/or the application of HE4 in the management of BC. One of the main limitations of this study is that it did not use a random sampling method; this means that the patients were chosen in order to acquire all the stages and degrees of BC. In addition, the study was conducted with a relatively small sample size, which may reduce the statistical power. Despite the limitations, since the current research was conducted as a preliminary study, it could be a valuable source for further research in the field. Investigating the biological functions of HE4 can lead to the discovery of basic molecular mechanisms responsible for HE4 role in the progression, invasion, and metastasis of BC. Also, it may help to clarify the events that lead to HE4 upregulation in cancer cells as well as designing new therapeutic strategies by targeting HE4 in order to develop a more effective treatment for BC.

## Conclusion

The present study confirmed that the increase in HE4 expression is probably associated with invasive phenotype of BC. According to the poor effectiveness of current treatment methods, HE4 may be an independent prognostic factor for poorly differentiated BC, and it may help identify high-risk BC patients. This can help BC patients benefit from a more aggressive adjuvant therapy. Our results also suggested that the measurement of plasma HE4 expression may be useful as a screening test for early detection of BC patients from healthy individuals in the early stages, as well as predicting cancer recurrence. It can be argued that in addition to HE4 biomarker capacities, it may also serve as a potential therapeutic target for inhibiting metastasis and cancer recurrence.
